# On Mental Diseases and Their Legal Relations

**Published:** 1845-01

**Authors:** 


### ART. III.

1. *Die Psychischen Krankheiten, und die damit verwandten Zustände in Bezug auf die Rechtspflege.* Von J. H. HOFFBAUER.—*Berlin*, 1844.

*On Mental Diseases, and their Legal Relations.* By J. H. HOFFBAUER. —*Berlin*, 1844. 8vo, pp. 266.

2. *A Treatise on the Medical Jurisprudence of Insanity.* By J. RAY, M.D., Superintendent of the Maine Insane Hospital. Second Edition, with Additions, &c.—*Boston*, 1844. 8vo, pp, 490.

3. *Remarks on the Impunity of certain attempts to Murder.* By T. MAYO, M.D. (*Medical Gazette*, 1843-4.)

THE first of these works is another of the numerous treatises on the medical jurisprudence of insanity, which of late years have issued from the German press. In his preface, the author states that his chief object is to remove the obscurity which has been thrown on the subject by the want of correct definitions and the improper application of the same term to very different states of the mind. He alludes to some errors of this kind in the well-known treatise of J. C. Hoffbauer, which has for many years been deservedly regarded as a standard work of forensic psychology.*

It is not our purpose to enter into those differences, which appear to us to be rather philological than medical. We apprehend that it is a matter of very little importance to English medical jurists, to deter-

* Die Psychologie in ihren Hauptandwendungen auf die Rechtspflege, von J. C. Hoffbauer, 1823.

mine the precise significations of the terms "*verstand*" and "*vernunft*," or the differences between "*wahnsinn*" and "*verrücktheit*;" and, practically speaking, we cannot perceive that the author of the present treatise has at all impugned the value in a medico-legal view of J. C. Hoffbauer's 'Psychologie.' With every desire to appreciate the labours of our German brethren in this department of science, it has always appeared to us, that they have been too prone to take up the metaphysical side of the question; and to leave those practical points, which are so deeply interesting to the medical jurist, in as much obscurity as ever. This fault exists, in our opinion, in the treatise before us. Thus discussions on the soul, free-will, and the "indivisible unity" of man are, in our judgment, quite misplaced in a medico-legal treatise. If the work of legislation were to begin anew, and legislators were willing to be instructed upon subjects, in relation to which they might with reason consider themselves to be as capable of forming a judgment as a professional man, there would be perhaps some ground for introducing such matters. But it is well known that medical witnesses are not required to show themselves versed in metaphysical speculations; and they are only likely to become confused by the introduction of them into medico-legal treatises on insanity.

In treating the subject, Dr. J. H. Hoffbauer shows himself to be a strict adherent to system. There are some advantages in this, especially for reference; but it is a question, whether it is not carried to too great an extent. At the first view, his arrangement appears too artificial for practice. The same main divisions of the subject are adopted which are to be found in most modern treatises on insanity; but there are numerous subdivisions, the necessity for which is questionable. English writers have generally contented themselves with giving simply the *legal* relations of each form of insanity; but the author treats in distinct sections, the civil, criminal, and police relations of each of the forms; thus we have in their appropriate sections, the police-relations of melancholia and somnambulism!

The author contends that in disputed cases it should rest with the medical practitioner alone to aid the court in its judgment, (16;) but he requires that the witness should be qualified for this duty to a degree which would certainly render most medical witnesses incompetent. Thus he should be both "a theoretical and practical psychologist, and a man of knowledge and extensive experience." In criminal cases in this country, it is a common practice to require the opinion of the medical practitioner who happens to be summoned as a witness respecting the cause of death. Sometimes an individual "de circumstantibus," volunteers his evidence as a member of the medical profession; and this is received and acted upon, when in neither case may the medical witness have paid the least attention to the medico-legal relations of insanity. Although we cannot go to the extent indicated by our author, as to the qualifications of witnesses, we have no hesitation in condemning the loose and inconsistent practice often pursued in our own courts of law. We hope to see a better plan adopted; and that the selection of medical witnesses to testify to the state of mind of a criminal, will not be left as it now is, to mere accident or to an interested selection made by the solicitors for the prosecution and defence.

We consider it unnecessary to enter into any further analysis of this work. It is not adapted to English practitioners, and will probably only find readers among those of our countrymen who have specially devoted their attention to the medico-legal relations of insanity.

The first edition of Dr. Ray's work on the 'Medical Jurisprudence of Insanity' was reviewed in No. XIX of this Journal. We then had occasion to speak well of it, as embracing sound and practical views, and we see no reason to alter our opinion in respect to this edition. It is written in very clear and intelligible language, and is well fitted to serve as a manual for the medical jurist. The author we observe still retains Foderé's incorrect version of the case of Fish *v.* Palmer, (p. 58 ;) and we find that after having read the correct report of the case of Miss Bagster, to which in our former notice we referred him, he adopts the conclusion which we then ascribed to him by inference, that this young lady "was really imbecile to a degree requiring legal interference (p. 92.) In this particular case, the only two independent witnesses, Drs. Haslam and Morison, who were summoned by the commissioners, and not by the parties interested in the result, gave a strong opinion that this person was not insane, but that her incompetency to manage her affairs arose from her youth and ignorance! We cannot object to Dr. Ray entertaining a different opinion, but we do think it somewhat inconsistent, that he should at the same time censure what he calls "the habitual practice of Great Britain at the present day." "One finds it difficult to believe," he remarks, on "what slight grounds interdiction is there every day procured; a measure that with the ostensible purpose of protecting the interest of the insane party is too often in reality designed to promote the selfish views of relations and friends." (p. 462.) We believe that there are few modern cases to which these remarks can be applied with greater propriety than to that of Miss Bagster; but in respect to this case, and in opposition to the strong opinion of the only two medical witnesses examined at the inquisition, whose evidence could be supposed to be entirely free from bias, he has no hesitation in concluding "that the verdict was correct." (p. 92.) We think it would be difficult to adduce any modern instance in which interdiction was procured in England upon slighter grounds than existed here.

It cannot be disputed that the criminal law of England is loose and vacillating in respect to individuals charged with crime, and for whom insanity is pleaded in exculpation. We do not think the author overstates the case in relation to this question when he says:

"If we carefully examine the cases tried within the last hundred years, as they are brought together in the various treatises on lunacy and on criminal law, the utmost respect for authority will not prevent us from observing the want of any definite principle as the ground of the difference of their results. Amid the mass of theoretical and discordant speculations on the psychological effects of insanity, and of crude and fanciful tests for detecting its presence, which these trials have elicited, the student who turns to them for the purpose of informing his mind on this branch of his profession will find himself completely disheartened and bewildered. Instead of inquiring into the effect produced by the peculiar delusions of the accused on his ordinary conduct and conversation, and especially of their connexion with the criminal act in question, the courts in these cases have been contented with laying down metaphysical dogmas on the consciousness of right and wrong, of good and evil, and of the measure of understanding still possessed by the accused. One principle after another has been successively abandoned, and resumed either with the strongest disregard of consistency, or the most extraordinary ignorance of previous decisions. Thus the old maxim that insanity does not annul criminal responsibility in one who retains the power of distinguishing right from wrong, was abandoned in the case of Hadfield, reaffirmed in that of Bellingham, again abandoned in the trial of Martin, subsequently modified by Lord Lyndhurst; and again in the year 1837, a jury holding in their hands the life of a fellow-man, are told by Mr. Justice Park, that as regards the effect of insanity on responsibility for crime, it is merely necessary that the party should have sufficient knowledge and reason to discriminate between right and wrong. (Greensmith's Case, No. xix, p. 144.) Three years afterwards, on the trial of Oxford for shooting at the Queen, Lord Denman told the jury that the question for them to decide was, whether the prisoner was labouring under that species of insanity which satisfied them that he was quite unaware of the nature, character, and consequences of the act he was committing; in other words, whether he was under the influence of a diseased mind, and was really unconscious, at the time he was committing the act, that it was a crime. On the trial of M'Naughten, for killing Mr. Drummond, in 1843, Lord Chief Justice Tindal instructed the jury that before convicting him they must be satisfied that, when committing the criminal act, he had not that competent use of his understanding as that he knew that he was doing a wicked and wrong thing—that he was sensible it was a violation of the law of God and man. Soon after the trial of M'Naughten, who was acquitted, the English House of Lords propounded certain questions, relative to the responsibility of the insane, to the judges of the law-courts; and their reply was meant, no doubt, to be considered as the law of the land, by which all future practice is to be regulated. They say that, to render the party irresponsible, 'it must be clearly proved that, at the time of committing the act, the party accused was labouring under such a defect of reason, from disease of the mind, as not to know the nature and quality of the act he was doing; or, if he did know it, that he did not know that he was doing what was wrong.' How this question is to be settled, and what is to be done with those cases where it is impossible to ascertain the views of the party, touching the moral quality of his acts, we are not told." (p. 48.)

We have quoted this passage because, in our opinion, it represents the actual state of the law of England. Many have been led to suppose that the answers returned by the judges placed this important question on a certain and well-defined basis; but substantially the answers left the question much as they found it, and judicial decisions hereafter are likely to be just as uncertain as they have hitherto been. (See No. XXXI, p. 273.)

We think Dr. Ray might have presented an analysis of the important cases of Oxford and M'Naughten in this edition. In our judgment, they would have been more interesting to the profession, both in America and England, than some which he has extracted from foreign works. The last chapter in the work, on Interdiction and Isolation, contains excellent practical remarks, with some additional matter. Indeed the whole subject is very judiciously treated, and we have no hesitation in recommending Dr. Ray's work to our readers as one of the most useful and practical on the medical jurisprudence of insanity.

Dr. Mayo's "remarks" are confined to the much agitated question of what should constitute irresponsibility in criminal cases, more especially in charges of murder. The author advocates a doctrine which, owing to the acquittals that have recently taken place on the alleged ground of insanity, for certain heinous crimes, is now becoming prevalent among those whose views are not obscured by the metaphysical speculations of German psychologists, namely, that there may be a degree of insanity in which punishment will operate as a preventive of crime, as much so as in respect to criminals who are undoubtedly sane. After stating that the insane are largely subject to fear, and that, in many instances, they are capable " of controlling, from prudential or other motives, their own tendencies;" he argues that " the power which, under the improved management of the present age, is so much employed for their benefit, and often for their cure, might, in these instances, be exhibited, in so far limiting their delusions, as to render them compatible with the peace of society." (p. 332.) At present we wait until they have committed some murderous act, and then inclose them in a madhouse for life. In this case, " the state of the alleged lunatic has all the attributes of a most bitter punishment, except those which tend to prevent the repetition of the offence by others. Soon, indeed, he is shut out from the thoughts and recollections of man, or if recollected by those who ought to profit by his sufferings, he is only recollected as one who has plenty to eat and drink and nothing to do." Thus, then, the psychological philanthropist, in carrying out his doctrine to an extreme degree, actually leads to the infliction of a severe punishment on the individual, but prevents it from having the least effect upon others as a public example! There can be but little doubt, that these doctrines are closely connected with capital punishment, and it is highly probable that, if this were abolished, nothing more would be heard of the plea of insanity in criminal cases.

Dr. Mayo appears to us to take a very sensible view of this question, in adopting, as he does, the maxim " in medio tutissimus ibis." He observes, in relation to the common legal test—of the individual being ignorant of the criminality of the act of murder at the time of its perpetration, that " the punishment of an insane person, whose insanity has proceeded to this point, can in no degree answer the intention of punishment. It cannot prevent a repetition of the offence, for it will not be appreciated by the persons whom it is supposed to influence." (p. 302.) This state would include those cases which depend on delirium, and it would embrace all such acts " as, springing out of an insane delusion, would be justifiable, if that delusive impression were founded in reality." A man, however, may know that his act is contrary to law, and be fully aware of its criminality ; yet he may *not* be " one whose act the threat of punishment could have prevented in him, or the example of whose punishment could deter others similarly situated." This is a fact familiar to medical jurists. Dr. Mayo mentions, as a striking instance, that " Hadfield himself apparently shot at the king with a view to being capitally punished for it."

The author agrees with the late dictum of our judges, that the proof of partial insanity should not necessarily carry with it irresponsibility for crime. In this respect he differs from Georget and Prichard. In considering the "*justice*" of inflicting punishment upon monomaniacs, or upon those who labour under partial delusion, he contends, that " the person who can reason upon his crime, who can devise systematic means for accomplishing it, who can form and weigh opinions respecting his chances of escape, who can conduct the ordinary business of life, and can take care of his property, cannot, consistently with justice, be made less responsible morally (legally?) than the unhappy offspring of the prostitute or thief," who, after running through a career of vice and crime, is brought to the gallows. With respect to whether the punishment will act as "a *preventive*," he contends, and we think fairly, that "the burthen of disproof must rest with those who undertake to deny this supposition." One fact appears to us certain, that if such assassinations as that of Mr. Drummond were to become frequent in our public streets, and the evidence of insanity, adduced to shield the perpetrators, were no greater than it was in that case, the legislature would be compelled to try the effect of a severe punishment as a preventive; for the safety of society is not to be compromised or continually put in jeopardy, merely for the sake of carrying out certain psychological theories.

Dr. Mayo condemns the decision in M'Naughten's case, because, admitting he was a monomaniac, there was no proof, 1, that he was not cognizant of the nature of his act, in reference to the laws; and 2, allowing him every benefit for a preexistent delusion, he could not have construed the circumstances under which he performed the fatal act as affording grounds for it on the plea of self-defence under an *immediate* attack. Admitting that he had one set "of morbid associations of thought, he was not, therefore, incapable of learning or of teaching others by the example of his punishment; that a strong desire of vengeance, or if you please of prevention of mischief, must not be gratified in defiance of the laws of the country. To say that he was incapable of making these distinctions would be to assign to him a pathological state, of which no evidence was adduced. He must have been a maniac, not a monomaniac." (p. 304.)

It appears to us impossible to deny the correctness of this inference; and the view is so far confirmed by the late "dicta" of all the judges, that if consistently carried out, a case like that of M'Naughten is not likely hereafter to terminate in an acquittal. We shall presently adduce an instance in which the law has been thus rigorously enforced.

The author looks upon the entire escape of Oxford as confessedly most mischievous. Among those writers who are extreme advocates of "irresponsibility," we have never yet met with one who could, with any show of reason, justify, on psychological grounds, the acquittal of Oxford and the conviction of Francis. The result, in the latter instance, can only be referred to the fact, that our law authorities began to doubt the propriety of the lenient verdict in the former case. "I am not," observes Dr. Mayo, "advocating severity of punishment, but merely demanding that the consequence of offences against the community, committed by the insane, when the punishment, primâ facie awarded by law, appears unfit, as revolting to our sense of justice, may, in certain cases, assume to them the character of punishment in some form or other, and not be wasted as at present in unproductive suffering."

The practical questions, in Dr. Mayo's opinion, connected with the plea of insanity in criminal cases, are these: 1, Whether the insane offender is aware, at the time of commission, of the nature of his act in reference to law, if not, the plea should undoubtedly be admitted; and 2, Whether, though aware of the illegality and criminality of the act, he is in a condition to be withholden by that knowledge from the offence; in which case the same ultimate grounds of exculpation should be admitted. "In each case, a state of *present* insanity *connected with the criminal act*, and rendering the threat of punishment inoperative, constitutes the plea in behalf of the offender. (p. 335.) We have some doubt how far it is correct to couple these reasonable conditions, with proof of the insanity being connected with the criminal act. Are we, as sane individuals, always in a position to be able to connect the delusions of the insane with their acts? The author objects to the practice of allowing medical witnesses to express an opinion, as to whether the accused is a "responsible or irresponsible agent." He contends with propriety that the medical evidence should be confined to the "pathology" of the case, to the condition of the man's mind, and that it has nothing to do with the question, whether the party be a "responsible or irresponsible agent." This is wholly beyond the province of the witnesses.

We have thus examined the opinions of Dr. Mayo, not merely because they are the most recent, but because they are reasonable, and have a direct practical bearing on this important subject. Since the case of M'Naughten, from thirty to forty trials for heinous offences have taken place in this country, in which the plea of irresponsibility has been raised. This shows that it is still a matter of deep importance to the English medical jurist. It is, however, a subject of regret to find that the complaint of Dr. Ray, of the vacillating character of our law, is fully borne out by the results. At one time, the legal standard of "right and wrong" is applied; at another, it is abandoned; in one case, the absence of motive is a ground of exculpation, at another it is not; in short, there is no consistency whatever. Some are acquitted, and others are executed, without our being able medically speaking, so far as this plea is concerned, to discern any marked difference in the cases.

It is not our purpose to prolong this article by a detail of cases, but still since the trial of M'Naughten, three of a remarkable kind have occurred and which are, therefore, deserving of a slight notice.

At the last Derby Winter Assizes, a young man named Grocock, was indicted for violating the person of a girl, aged 11 years, and also for attempting to murder her. The prisoner decoyed the girl to a solitary place under the pretence of giving her employment; he violated her person, then struck her repeatedly on the head with a hammer, and left her for dead. He then gave himself up to justice as a murderer, asked for pen, ink, and paper, and wrote down a minute account of the crime which he had committed, excepting the rape, stating in conclusion, "as regards my intention for committing such an act, I was determined to be transported or hung, having at that time no means of obtaining a livelihood, but I cannot properly explain the motive for committing such an action." In prison he was calm and quiet, when he did not know he was watched, but rolled his eyes strangely when he knew that he was observed. It did not appear that the prisoner thought the girl was a "conspirator" against him, but general evidence was adduced to show that he had previously complained "of feeling curious,—that his head was hot," and he felt as if he should go "beside himself." One witness thought him wild in his conversation and appearance, and it was proved that a near relative had been confined as a lunatic for eighteen years. There was no medical evidence of insanity, and the prisoner was convicted and sentenced to transportation for life.

If we try this case by the criteria of homicidal monomania, laid down by Georget and Prichard, it would appear that this criminal should have been acquitted. 1. There were peculiarities of conduct in the individual; weak, it is true, but still as strong as in Oxford and M'Naughten's cases. 2. The acts were without motive. 3. He sought no escape, delivered himself up to justice, and acknowledged his crime. 4. He had no accomplices. This case shows that such criteria are insufficient to establish a plea of irresponsibility, for no one can doubt that the prisoner was very properly convicted and punished.

Another remarkable case was tried at the Lewes Lent Assizes, 1844: The Queen *v.* Laurence: The prisoner had been arrested by a constable for a petty theft; he was taken to the police-station, where the inspector who was an utter stranger to him, was at the time engaged in talking to some friends, his back being turned to the prisoner. The man suddenly seized a poker and struck the inspector a violent blow on the skull, from which he speedily died. The prisoner admitted that he struck the blow, that he had no motive for the act, and that he would have struck any one else who had been standing there at the time. He also said he hoped the deceased would die, he was glad he had done it, and he wished to be hanged. The evidence at the trial showed that there was no cause of quarrel between the parties, but that the prisoner appeared to be actuated by some sudden impulse, for which they could not give the slightest explanation. This man was left to a chance defence, for the Court was actually obliged out of common humanity to assign a counsel to him! There was no eloquent advocate to make a brilliant speech in his defence, no array of medical witnesses, versed in the subject of insanity, to contend for the existence of impulsive homicidal monomania, and thereon demand the man's acquittal as an "irresponsible agent;" but the defence was simply a cold formal plea of insanity, resting upon the fact of the deceased being a stranger to him, and there being consequently no motive for the act of murder. The jury negatived this plea, and the prisoner was convicted and executed.

We consider that this was one of those cases in which, in Dr. Mayo's view, an example may be properly made by punishment. The only points in which it substantially differs from M'Naughten's were these, that there was *less evidence of deliberation and stronger evidence of sudden impulse in the act;* and that there were no exertions used, in getting up the medical defence. Supposing that the party killed, had been some individual of high station in society, the probability is, that the result would have been different, and that Laurence would have now been spending his time with Oxford and M'Naughten. But it may be contended, this man was improperly convicted and punished. We do not think that this would be the general feeling of the public mind. Assassination attempted or perpetrated without apparent motive is now becoming too common; and, if it be alleged that the punishment of the perpetrators cannot act as a preventive, we are inclined to say with Dr. Mayo, that the proof of this rests with those who make the allegation. Every member of the social state has a right to have his life protected by the laws, and experience shows that punishment has a very powerful effect in restraining ill-disposed men from the crime of murder.

The recent case of Dalmas must be fresh in the minds of our readers. This man was convicted and sentenced to death for cutting the throat of a woman on Battersea bridge. Insanity was not pleaded, although the crime was not only committed without motive, but against all common human motives. The prisoner has been reprieved on account, it is said, of some doubt respecting the proof of his identity. This being, of course, a legal point, does not require consideration, further than admitting the existence of the doubt, the prisoner ought to be discharged as an innocent man. But before the reprieve, he was frequently visited and examined by two medical witnesses, whose report is said to have led to the suspension of the sentence, on the ground that the prisoner was insane. Admitting this assumption, although not the slightest proof of this condition was adduced at the trial, the prisoner resting his defence simply on the ground that he was not the party who committed the crime, (a very unusual plea, in cases of real homicidal monomania,) he should be confined as a lunatic; but it is alleged that the sentence has been commuted to transportation for life. If this be carried out, it is hard to conceive how the question of insanity could be entertained. The results of these trials of Laurence and Dalmas appear to show that, in one case, where insanity is actually and with some plausibility pleaded, no pains are taken to make a searching inquiry into the justice of the plea; but that where insanity is not pleaded, and the commission of the crime is wholly denied by the criminal, then an investigation is directed to be made by medical men into the state of the convict's mind. Such a mode of administering the law requires no comment.

---

